# Impact of Mild Traumatic Brain Injury on Sleep Patterns in Younger Individuals: A Systematic Review

**DOI:** 10.1177/2689288X251377029

**Published:** 2025-09-15

**Authors:** Sid Subramanian, Caitlin M. Carroll

**Affiliations:** ^1^ Department of Psychiatry and Behavioral Medicine, Wake Forest University School of Medicine, Winston-Salem, North Carolina.; ^2^ Section on Gerontology and Geriatric Medicine, Department of Internal Medicine, Wake Forest University School of Medicine, Winston-Salem, North Carolina.

**Keywords:** actigraphy, mTBI, polysomnography, sleep, sports concussion

## Abstract

Concussions or mild traumatic brain injuries (mTBI) are a frequent outcome of contact sports, and adolescents and young adults face a disproportionately higher risk of repeated injury. Prominent sequelae of mTBI observed in younger populations include anxiety, decreased sleep quality, insomnia, and impaired cognitive performance, all of which can extend beyond the acute phase of recovery. Although sleep is an essential aspect of the recovery process, few studies have evaluated the impact of mTBI on objective sleep measures, especially in younger athletes. The purpose of this systematic review was to analyze current literature on objectively measured sleep following mTBI. Following Preferred Reporting Items for Systematic reviews and Meta-Analyses (PRISMA) guidelines, out of 2154 records, 17 studies were included, focusing on individuals aged 10–30 years, as they experience more sports-related concussions. Across 15 actigraphy and 2 polysomnography studies, results concerning total sleep time and sleep efficiency were mixed. However, several studies observed increased wake after sleep onset, a marker of sleep fragmentation, throughout mTBI recovery. Increased sleep fragmentation may explain frequently reported sleep complaints in this population and could ultimately contribute to a cycle of increasingly disrupted sleep. Additional research is needed to understand the relationship between mTBI and sleep, as sleep disruption during this critical period of neurocognitive development can have long-term impacts on brain health.

## Introduction

Mild traumatic brain injury (mTBI), or concussion, represents a growing public health concern nationwide, with between 1.7 and 3.8 million cases reported each year.^1^ mTBI accounts for roughly 90% of all TBI cases in the USA,^2^ and while they rarely lead to permanent disability, associated postconcussive symptoms can severely impact quality of life. These symptoms include headaches, dizziness, cognitive impairment, anxiety, depression, poor sleep quality, and daytime fatigue, all of which can persist for months to years after the initial injury.^3–8^ Particularly relevant to younger populations, more severe symptoms are associated with greater school-related problems and daytime dysfunction.^9^ Moreover, adolescence, generally considered ages 10–19 years, is a period of extensive neurocognitive development where prolonged symptomology may have long-term consequences on brain health.

To date, most studies of mTBI focus on older combat-exposed veterans; however, there is increasing concern about the impacts of mTBI on younger populations given their participation in contact sports.^10^ Adolescence is a critical period for both brain development and changes in sleep–wake patterns; therefore, brain injury during this time can have detrimental impacts on neurocognitive development.^11,12^ Adolescence is marked by increased white matter volume (myelination) and a correlated decrease in gray matter volume (synaptic pruning). These structural changes are associated with advances in executive functioning, cognitive processing speed, and memory plasticity.^13–16^ Concurrently, there are large shifts in objectively measured sleep–wake characteristics. There is a significant decrease in slow-wave sleep, the deepest, most restorative aspect of non-rapid eye movement (NREM) sleep. Adolescents also typically have a delayed circadian rhythm, with progressively later sleep onset and shorter sleep durations.^17–19^ Further, quantitative electroencephalography (EEG) analyses, which break down the specific brainwave patterns that occur during sleep, show decreased absolute power, or overall brain activity, reflecting synaptic pruning.^20^ There are also distinct shifts in the location of slow-wave activity, the main brainwave of slow-wave sleep, within the brain. Frontal slow waves tend to increase during development, mirroring the cortical maturation of frontal brain regions.^21–25^ Sleep spindles, a distinct feature of Stage 2 NREM sleep, appear as a burst of faster frequency activity on an EEG. Sleep spindle frequency increases across adolescence and is associated with age-related improvements in cognitive performance and memory.^26^ While there are clear connections between brain structural development, sleep, and cognitive function, there is little long-term data exploring how mTBI may disrupt sleep and cognitive processes.

Contact sports are a main source of mTBIs in youth, and athletes share a disproportionate burden of all mTBI cases.^27,28^ Unfortunately, many mTBIs are not reported, despite mounting evidence that repetitive head impact can disrupt blood–brain barrier function and lead to neurodegenerative disorders.^29–32^ This underreporting is likely due to a variety of factors, including poor access to healthcare, lack of perceived severity, and an unwillingness to miss time due to injury.^33,34^ Further, we lack standard objective measures for quantifying injury severity and calculating recovery timelines. This can increase the risk of subsequent injury, as those with a history of mTBI have a 3–6 times increased risk of experiencing subsequent concussions.^35–37^ Repeated concussions (rmTBI) are associated with severe sleep symptoms, such as insomnia, early awakenings, fragmented sleep, and daytime fatigue and dysfunction.^38,39^ Further, many studies show rmTBI is linked to the accumulation of pathology associated with chronic traumatic encephalopathy (CTE)^40,41^ and Alzheimer’s disease (AD).^42^ Sleep plays a significant role in the pathophysiology of both of these neurodegenerative diseases^43,44^; therefore, understanding the impacts of mTBI on sleep may be important for limiting the long-term consequences.

The pathology of mTBI is remarkably heterogeneous, and many symptoms are not predicated by imaging alone.^45^ Therefore, identifying objective metrics to quantify mTBI severity and evaluate recovery progress is essential. One potential objective measure is sleep, as it offers a powerful yet noninvasive proxy of transient disruptions in brain function, glucose metabolism, and axonal injury.^45^ Sleep abnormalities are one of the most commonly reported postconcussive symptoms, with 30–70% of people reporting sleep disturbances.^46^ Further, individuals who report sleep problems are more likely to report other postconcussion symptoms.^47,48^ Sleep loss may mediate other mTBI symptoms and slow recovery^49–51^ through the promotion of a proinflammatory environment.^19,49–54^ This is particularly important in young athletes with developing brains, as mTBI-related chronic inflammation is associated with long-term deficits in synaptic plasticity,^55^ disrupted neuronal connectivity,^56–58^ impaired cognitive performance,^59,60^ and neurodegeneration.^61,62^ Interestingly, poor preinjury sleep patterns are also correlated with more pronounced postconcussive neurocognitive and sleep-related deficits.^63^ Together, these data suggest sleep disturbances are both a symptom of mTBI and a driver of more severe mTBI symptomatology.

The purpose of this systematic review is to evaluate the current body of literature on mTBI and objective sleep metrics in young populations. To date, most studies looking at the interaction between sleep and mTBI incorporate only subjective measures of sleep, such as the Pittsburgh Sleep Quality Index or Epworth Sleepiness Scale. While informative, these methods are prone to bias and often fail to accurately reflect objective changes in sleep physiology.^64^ Objective methods of measuring sleep, including wrist-based accelerometry or polysomnography (PSG), provide a more accurate picture of sleep architecture, total sleep time (TST), and sleep fragmentation. PSG is particularly useful, as it provides insights into sleep microarchitecture or patterns of neural activity across different phases of sleep. These methods may allow for a more mechanistic understanding of the relationship between sleep and mTBI, providing greater opportunity to identify avenues for intervention.

## Methods

### Protocol

**FIG. 1.**
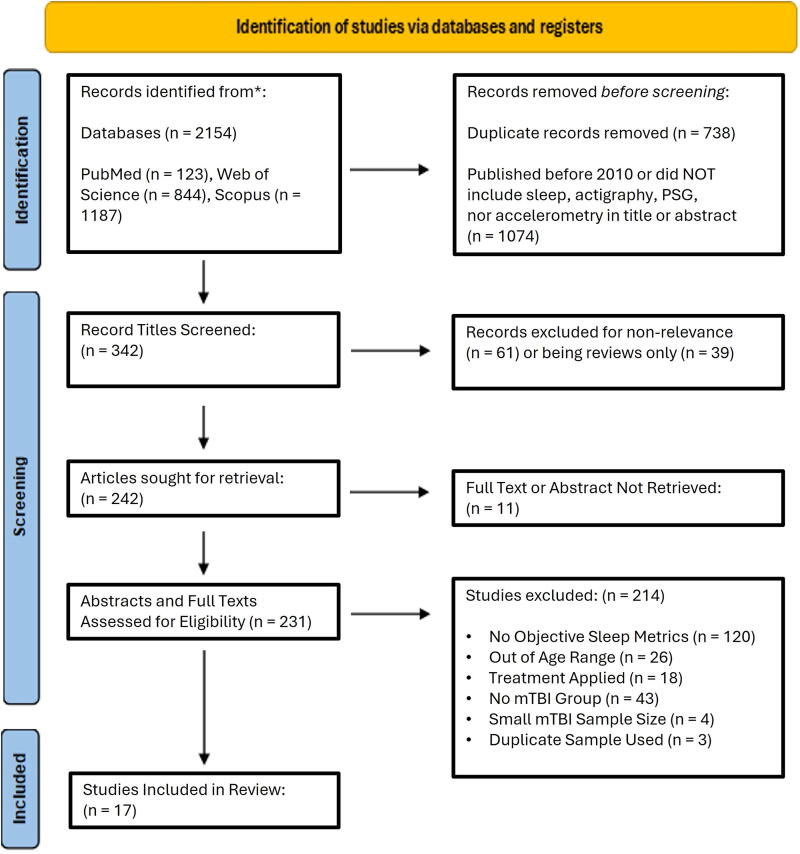
PRISMA flowchart of study retrieval and selection. From 2154 identified studies via 3 databases, 342 titles were manually screened. Records excluded for nonrelevance included older adults, animals, or veterans, for example. The remaining 242 articles were evaluated in full-text form. Conference abstracts were included to prevent publication bias and offer additional data, considering the limited studies available in this population. The final step identified any remaining articles that failed to meet the inclusion criteria. For example, most articles with “no mTBI group” were reviews missed by a prior screen. Some studies that measured objective sleep metrics also incorporated subjective sleep metrics. However, studies with only qualitative questionnaires and self-reports were excluded after thorough assessment to ensure no negative findings were obtained via actigraphy or PSG. Studies without a control or non-mTBI group were considered for analysis if other criteria were met. mTBI, mild traumatic brain injury; PSG, polysomnography.

This systematic review followed PRISMA guidelines.^65^ An initial query was made in August 2024 using 3 databases (PubMed, Web of Science, and Scopus) to identify records (EndNote^TM^ 21). The main keywords were [“sleep” AND “mTBI”], [“sleep” AND “concussion”], and [“sleep” AND “mild traumatic brain injury”] in any field, such as title, abstract, or full text, if accessible. All records were further screened for a publication date of 2010 or later. This step was included to narrow our focus on recent advances in the field. The next screening step was for [“sleep”] in the title or [“polysomnography OR “actigraphy”] in any field. This step was added to identify papers using objective measures of sleep and ensure that studies where sleep was not a primary outcome were still properly identified. Conference abstracts were included to mitigate positive result publication bias,^66^ although if conference abstract data were duplicated in a later publication, the full publication was chosen instead. Those records are shown as “duplicate sample used” in Figure 1. Additional screening steps involved removing records with the search words of [“review”] or records with any of the following terms in the title: [“veteran” OR “warfighter” OR “combat” OR “older adult” OR “mice/mouse/rat”]. The remaining articles were evaluated in either conference abstract or full-text form. Articles that employed no objective metrics of sleep (actigraphy, EEG/PSG) were eliminated. Further, articles that did not have an mTBI group, clinical case reviews, and intervention studies were excluded. We focused on studies in which the majority of participants were 10–30 years old, as shown in Figure 1. Given the current literature, we found it necessary to expand the age range beyond just the clinical definition of adolescents, as many studies included college-age athletes or other young adults just beyond the clinical age range. We felt this was appropriate given that brain development continues through the mid- to late-twenties.^67,68^ While some veterans fit in the age range of the study, we focused solely on nonveteran populations to avoid the confounding and synergistic impacts that blast-related trauma and PTSD can have on sleep beyond the typical sequelae of mTBI.^69^ A summary of relevant criteria is provided below for clarification.

### Inclusion criteria

We included studies that (1) assessed mild traumatic brain injury (2) using overnight actigraphy or EEG/PSG techniques, (3) and had full texts or abstracts available online.

### Exclusion criteria

We excluded studies that (1) focused on veteran populations, (2) incorporated only questionnaires or other subjective metrics of sleep, (3) included participants primarily outside of the age range of 10–30 years, (4) contained an intervention, (5) were clinical case reports, (6) were published before 2010, or (7) were not agreed upon by S.S. and C.M.C., were excluded.

### Data processing and analysis

For each selected article, relevant parameters were extracted and confirmed by S.S. and C.M.C., as shown in Table 1. These include the age range of the mTBI and control groups, sample sizes, time since injury, and objective sleep metrics measured. We included Stevens et al. (2023)^84^ despite the large reported age range of 20–52 (mean age = 24) because the study incorporated only sports-related concussions and used PSG, making it valuable for inclusion in the analysis for comparison purposes. In Table 2, the mean values of all sleep metrics were compared across studies. If a study looked at children and adolescents separately, results from young children (age <10 years) were not considered. The standard deviation or a confidence interval was included if available. For studies where measurements were averaged on a weekly basis, the most recent (proximal to time of injury) weekly average was included. In Table 3, we compared both subjective and objective sleep metrics between mTBI and control groups in 11 studies that included a control group.

**Table 1.** Summary of Selected Articles on mTBI in Adolescents and Young Adults

Author (year)	Methods used	mTBI group (*n*)	Control (*n*)	Age (SD)	Time since injury	Study duration
Tham (2015)^11^	Actigraphy	50	50	15.9 (2.0)	3–12 months	10 days
Raikes (2016)^70^	Actigraphy	7	10	24.05 (1.88)	<1 week	Two 5-day sessions
Mantua (2017)^3^	PSG	16	14	19.9 (1.5)	1–10 years	1 night
Sufrinko (2018)^71^	Actigraphy	19	NA	15.4 (2.0)	<3 days	1–4 weeks
Lindsey (2019)^72^	Actigraphy	5	5	21 (2.9)	<3 days & >2 months	5 days
Tham (2019)^73^	Actigraphy	28	NA	13.96 (1.83)	<7 days	3 weeks
Barlow (2020)^74^	Actigraphy	83	25	13.0 (0.5)	4–6 weeks	5–7 days
Hoffman (2020)^75^	Actigraphy	17	NA	20 (1.5)	<3 days	Until symptoms resolved
Maerlender (2020)^76^	Actigraphy	11	9	13.4 (1.6)	<2 weeks	1 night
Wilson (2021)^77^	Actigraphy	32	NA	15 (1.7)	<2 weeks	6 night
Considine (2021)^78^	Actigraphy	57	26	18.0 (1.44)	1–2 days	2 weeks
Trbovich (2021)^79^	Actigraphy	17	NA	15.35 (2.09)	<3 days	3 weeks or symptom resolution
Bone (2022)^80^	Actigraphy	7	20	19.93 (1.14)	Not provided	7–10 days or symptom resolution
Fisher (2022)^81^	Actigraphy	55	Yes^a^	15 (12–18)^b^	<4 weeks	4 weeks
Crowley (2023)^82^	Actigraphy	11	11	NCAA Division III Athletes (∼18–22)	<4 days	1 week
Neely (2023)^83^	Actigraphy	49	NA	14.7 (1.9)	<2 weeks	2 weeks
Stevens (2023)^84^	PSG	10	Yes, Normalized^c^	24 (20–52)^d^	<1 week & >8 weeks	2 nights

^a^ Control values were derived from a systematic review/meta-analysis of pediatric nighttime actigraphy sleep parameters using pooled mean estimates from 79 studies. Mean control estimates were given based on age bins, and Fisher (2022)^81^ incorporated 2 groups: *n* = 24 children (5–11 years old) and *n* = 55 adolescents (12–18 years old). Single-sample *t*-tests were used for comparisons between study and control parameters. Adolescent TST was compared with the 15–18 y/o bin parameter. SE, WASO, and SOL control parameters were only available for the range of 3–14 y/o or 3–18 y/o and were not broken down into more specific age classifications in the control meta-analysis. Therefore, the control estimate used for comparison in Fisher (2022)^81^ was derived from participants outside of the adolescent age range.

^b^ An age range is listed (12–18 y/o), as the main study in Fisher (2022)^81^ included a sample of 24 children and 55 adolescents with a total mean age of 12.5 years old (5.96, 17.73).

^c^ Normative values were derived from a calculator that distinguishes by sex, age, and first or subsequent night. Parameters were modeled from a large-scale systematic review and meta-analysis that analyzed 169 studies and 5273 healthy control participants in overnight level 1 in-laboratory sleep studies.

^d^ Reasons for inclusion of Stevens (2023)^84^ previously described in **METHODS**.

mTBI, mild traumatic brain injury; SE, sleep efficiency; WASO, wake after sleep onset; SOL, sleep onset latency.

**Table 2.** Sleep Parameter Values for mTBI Participants

Study	Developmental stage	Postinjury phase	TST ± SD (min)	WASO ± SD (min)	SE ± SD (%)	SOL ± SD (min)
Tham (2015)^11^	Adolescence	Chronic	353 ± 58	112 ± 54	75 ± 11	—
Raikes (2016)^70^	Young Adult	Acute Subacute	435 ± 48 402 ± 86	48 ± 35 68 ± 62	88 ± 6.5 83 ± 12	—
Mantua (2017)^3^	Young Adult	Chronic	366 ± 51	28 ± 15	88 ± 6.2	19 ± 17
Sufrinko (2018)^71^	Adolescence	Acute	424 ± 41	—	83 ± 8.1	—
Lindsey (2019)^72^	Young Adult	Acute	640^a^	—	95^a^	—
Tham (2019)^73^	Adolescence	Acute	460 ± 77	—	87 ± 4.6	—
Barlow (2020)^74^	Adolescence	Subacute	477 ± 17	38 ± 1.4	83 ± 0.8	20 ± 1.7
Hoffman (2020)^75^	Young Adult	Acute	380 ± 89	—	74 ± 8.9	13 ± 10
Maerlender (2020)^76^	Adolescence	Acute	477 ± 70	—	96 ± 3.5	46 ± 19
Wilson (2021)^77^	Adolescence	Acute Subacute	432 ± 48 420 ± 60	—	—	—
Considine (2021)^78,b^	Adolescence/Young Adult	Acute	356 ± 116	—	92 ± 3.3	—
Trbovich (2021)^79^	Adolescence	Acute/Subacute	414 ± 48	—	84 ± 7.7	—
Bone (2022)^80^	Young Adult	Acute	286 ± 87	—	—	—
Fisher (2022)^81,c^	Adolescence	Acute Subacute	462 ± 12 468 ± 9.8	114 ± 6.2 108 ± 5.9	77 ± 1.1 79 ± 0.9	—
Crowley (2023)^82^	Young Adult	Acute	—	65.9	—	—
Neely (2023)^83,d^	Adolescence	Acute	450 ± 42	—	—	—
Stevens (2023)^84,e^	Young/Middle-Aged Adult	Acute Subacute	466 (396–535) 479 (427–561)	46 (21–63) 25 (20–29)	89 (86–91) 93 (92–95)	14 (5.8–21) 10 (7.3–13)

For each study, the following mean sleep parameter values were extracted for concussed participants: TST (nighttime), WASO, SE, and SOL. Since all studies used at-home measurements, actigraphy and polysomnography measurements were both included. The stage represents a broad classification of participants’ ages, with adolescents ranging from 12 to 19 years old, and young adults ranging from 20 to 30 years old. Some overlap was observed between categories, but most participants in each study fell within 1 designated category. The time intervals of acute, subacute, and chronic represent the time since the concussion. Measurements taken only in the first 2 weeks were deemed as the acute phase of recovery, and from 2 weeks to 2 months as subacute. Beyond 2 months served as the cutoff for the chronic phase of recovery. Studies without exact values for sleep parameters were excluded from this figure. For simplicity, values >10 were rounded to the nearest integer.

^a^ No standard deviation values were provided in the study.

^b^ All estimates were taken from average TST and SE 4–7 days postconcussion (most-acute measurement provided).

^c^ Data were available as averages for weeks 1–4. For simplification, the acute measurement was taken as the week 1 average, whereas the week 4 average represented the subacute measurement.

^d^ For Neely (2023)^83^, mean TST was taken from the group with persistent postconcussion symptoms, as the study separated mTBI participants into 2 groups: persistent symptoms present or not present.

^e^ Stevens (2023)^84^ did not provide standard deviations with estimates, but instead interquartile values, which are indicated next to the mean value.

mTBI, mild traumatic brain injury; SE, sleep efficiency; WASO, wake after sleep onset; SOL, sleep onset latency; TST, total sleep time.

**Table 3.** Comparison of Subjective and Objective Sleep Parameters Between mTBI and Control Participants

Study	TST	WASO	SE	SOL	REM (%)	REML	Fatigue	Sleep quality
Tham (2015)^11^	**↓**	**↑**	**↓**	—	—	—	—	**↓**
Raikes (2016)^70^	n.s	n.s	n.s	—	—	—	—	—
Mantua (2017)^3^	n.s.	n.s.	n.s	n.s	**↓**	**↑**	n.s	n.s
Lindsey (2019)^72^	↑^a^	n.s	n.s	n.s	—	—	—	—
Barlow (2020)^74^	n.s	n.s	n.s	n.s	—	—	—	—
Maerlender (2020)^76^	n.s	—	n.s	↑	—	—	**↑**	—
Considine (2021)^79^	n.s	—	n.s	—	—	—	—	—
Bone (2022)^80^	**↓**	—	—	—	—	—	—	—
Fisher (2022)^81,b^	**↑**	**↑**	**↓**	—	—	—	—	—
Crowley (2023)^82^	—	**↑**	—	—	—	—	—	**↓**
Stevens (2023)^84,c^	**↑, ↑**	n.s, ↑	n.s, ↑	n.s, ↓	↑, n.s	↑, n.s	—	—

Of the 17 studies, 11 included a designated control group, either via recruited participants or a meta-analysis/systematic review. The symbols indicate whether the metric in the mTBI group was significantly greater “↑”, significantly less “↓”, not significant “n.s”, or not measured “--” compared to the control group metric.

^a^ Because this was a pilot study, no significance was provided, but sleep differed by 200 min in mTBI versus control participants with a large effect (*d* = −1.90).

^b^ Fisher (2022)^81^ assessed children and adults separately. Only measurements from adolescents (12–18 y/o) were compared for TST, WASO, and SE.

^c^ Stevens (2023)^84^ gathered data twice, first in the acute phase (<2 weeks) and again in the subacute phase (<8 weeks). Significant differences are listed for the acute phase relative to normative controls and then the subacute phase relative to normal controls, separated by a comma.

mTBI, mild traumatic brain injury; SE, sleep efficiency; WASO, wake after sleep onset; SOL, sleep onset latency; TST, total sleep time.

## Results

### Section 1—study characteristics

A total of 17 studies were included in this systematic review, as shown in Table 1, including 14 full-length publications and 3 conference abstracts. Of the included studies, 15 contained wrist-worn accelerometry data and 2 contained in-home PSG recordings, but all studies reported some objective sleep metrics. Those measures included TST, wake after sleep onset (WASO: a measure of sleep fragmentation), sleep efficiency (SE: time asleep divided by total time in bed), and sleep onset latency (SOL: time to fall asleep). Most studies focused on the acute phase of recovery, which generally persists for 2 weeks postconcussion and is marked by the most severe symptoms, although some studies did report results from the subacute (4–6 weeks postinjury) or chronic phases of injury recovery (several months to years postinjury) (Table 1).^3,11^ Study duration also varied, with actigraphy studies reporting results between 1 day and 4 weeks and in-home PSG reporting data from either 1 or 2 nights.

### Section 2—general findings

TST was reported by nearly every study. TST estimates ranged from 286 to 640 min, although most studies fell into the range of 350–475 min, with adolescents tending to sleep longer, as expected. Of note, Bone (2022)^80^ reported an average TST of 286 ± 87 min, well below the other reported values (Table 2). Because it was a conference abstract, we were limited in our ability to explore factors contributing to this low TST. However, the study enrolled collegiate athletes, a population known to have low TSTs, which may partially explain this result.^85^ Lindsey (2019)^72^ reported the upper limit of 640 min of sleep per night; however, they did not report a standard deviation or offer a potential explanation for this amount of sleep.

In total, 10 studies compared TST between control and mTBI groups. Results were mixed, with 5 studies reporting no significant difference between groups, while 3 reported increased TST^72,81,84^ and 2 reported decreased TST^11,80^ (Table 3). Time since injury did not seem to have an impact on TST, as the postinjury interval was mixed among the results. For instance, Fisher^81^ reported increased TST in individuals 4 weeks postconcussion compared with controls in individuals. However, Barlow^74^ reported no significant difference in TST between mTBI and control groups using the same postinjury interval. Therefore, the variability in TST results may be due to baseline differences in TST between groups or other factors unrelated to injury. For instance, Bone (2022)^80^ reported an abnormally low TST in the mTBI group, which may reflect lower baseline TST in this set of athletes rather than any injury effect. Other factors such as school alarms or scheduling conflicts could have influenced TST in these groups, indicating we would need additional information about each population to draw conclusions.

Mean estimates of WASO, a measure of sleep fragmentation across the night, also varied between studies. WASO ranged from 25 to 114 min, with most studies reporting values between 25 and 65 min (Table 2). Insomnia is characterized by a WASO value exceeding 30 min more than 3 nights a week for at least 6 months.^86^ All studies reported WASO measurements above this nightly cutoff in the acute phase, suggesting severe sleep fragmentation through this phase of mTBI recovery (Table 2). This sleep fragmentation largely persisted through subacute and chronic phases of recovery, suggesting sleep disruptions may persist long term, thereby increasing insomnia risk.

Of the 8 studies that compared WASO between mTBI and control groups, 4 reported increased WASO for mTBI participants. Interestingly, Stevens (2023)^84^ and Tham (2015)^11^ reported persistent increases in WASO into the subacute and chronic phases of mTBI recovery, respectively. This suggests sleep fragmentation may be a long-term consequence of mTBI. While 4 studies did not find differences between control and mTBI participants, no study found decreased WASO in the mTBI group (Table 3), suggesting that WASO may be a more reliable metric for quantifying disrupted sleep in mTBI in multiple phases of recovery.

SE was reported in 13 studies, with values ranging from 74 ± 8.9% to 96 ± 3.5% (Table 2). SE is calculated as the time spent asleep divided by the total time spent in bed, with values <85% considered poor SE. Reported sequelae of long-term low SE are fatigue, irritability, and daytime sleepiness, and it is commonly observed in individuals with insomnia. SE is typically associated with WASO, where increased WASO decreases SE, although not all studies reported both metrics. In Tham (2015),^11^ for example, reported SE was 75 ± 11%, indicating impaired SE, which was expected given the WASO of 112 ± 54 min (Table 2). Similarly, mTBI participants in Mantua (2017)^3^ had a relatively high SE of 88 ± 6.2% and comparatively lower reported WASO of 28 ± 15 min. (Table 2). Overall, SE was relatively unimpaired across the different phases of recovery, with only 3 studies reporting values <80%. Of those studies reporting low SE, both Hoffman^75^ and Tham^11^ reported very high standard deviations. While this may accurately reflect variability in SE in their populations, it could also be due to technical difficulties in estimating SE from actigraphy or other factors such as low baseline SE. Of note, both PSG studies, which should more accurately detect SE, reported SE between 88 and 93.

When comparing SE between mTBI groups and control groups, no significant difference was observed in 6 of the 9 studies (Table 3). Fisher (2022)^81^ and Tham (2015)^11^ reported decreased SE in acute and chronic phases of recovery, respectively. Interestingly, Stevens (2023)^84^ found no difference in the acute phase but reported increased SE for mTBI participants in the subacute phase of recovery (Table 3). This result could indicate a rebound of SE during the subacute phase to promote brain recovery. However, these participants were performing in-home PSG for the second time. Therefore, the results may reflect an adaptation to the sleep recording process rather than improved SE, and future studies are needed to confirm these results. Overall, while some studies suggest poor SE is a sufficient marker of mTBI, future studies are necessary to clarify this relationship further.

Finally, sleep onset latency (SOL) was reported in 5 studies, ranging from 10 ± 3 to 46 ± 19 min. Most of these results fall around the expected SOL for this population of 10–20 min^87^ (Table 2), suggesting mTBI does not have a large effect on SOL. The only study that reported a mean SOL outside the expected range was Maerlender’s (2020)^76^ (46 ± 19 min). Maerlender’s was also the only study to report significantly increased SOL among those in the mTBI group as compared to the control group (Table 3). This discrepancy may be partially explained by the 1-night study duration. Because these actigraphy-based sleep metrics can be variable night-to-night, limited recording time makes it difficult to compare with studies recording for 5–7 nights. Stevens (2023)^84^ reported decreased SOL in the subacute phase, which might indicate increased sleep pressure or simply reflect the comparison of in-home PSG to in-lab PSG recordings, as participants likely felt more comfortable in their own homes. Further, because no study reported average bedtimes, it is difficult to interpret SOL results and determine if there were any mTBI-related circadian shifts. This is especially relevant for adolescent populations, as they are typically phase delayed.^19,88^ Together, these data suggest SOL is not an accurate sleep metric for measuring mTBI severity or recovery, although disruption may be present for some individuals.

For the PSG studies, we included a subset of sleep-staging parameters, focusing on rapid-eye movement (REM) (%) and REM latency (minutes) (Table 3). In Mantua (2017),^3^ participants who self-reported concussions between 1 and 10 years prior spent significantly less time in REM sleep and took longer to enter the first period of REM compared to age-matched control participants. Stevens (2023)^84^ similarly reported increased REM latency, but decreased time spent in REM only in the acute phase of recovery (Table 3). This difference may reflect a shift in the characteristics of REM sleep during mTBI recovery. While acute recovery may cause decreased time in REM, chronic recovery may feature a REM rebound, which is often considered an adaptive response to sleep deprivation.^89^

While only 4 studies compared subjective assessments of sleep quality and fatigue, we felt it was important to mention given the prevalence of this data in the literature. In Tham (2015),^11^ mTBI participants reported reduced sleep quality according to the adolescent sleep–wake scale, which aligns with the observed increase in WASO and low SE (Table 3). Similarly, Crowley (2023)^82^ noted an inverse relationship between WASO and sleep quality, although the questionnaire used was not specified (Table 3). Maerlender (2020)^76^ reported elevated fatigue levels in mTBI participants based on the Pediatric Quality of Life Inventory fatigue scale, which is often associated with increased SOL, consistent with what was reported in the study. Mantua (2017)^3^ reported no significant differences in subjective sleep; however, the extended time since injury may have affected those results (Tables 1 and 3). In general, subjective sleep reports seemed to be associated with the observed objective sleep metrics, but further studies are needed to confirm these relationships.

Although sex differences were not reported by every study, likely because of their relatively small sample sizes, there were some reported differences in several sleep metrics. Tham (2015)^11^ reported that males with mTBI had poorer SE compared to females. Sufrinko (2018)^71^ found that concussed male participants had increased TSTs compared with female participants. Fisher (2022)^81^ reported females had increased WASO and more arousals during concussion recovery. Given the sex differences in brain maturation over this time period, future studies should consider sex differences when evaluating sleep differences in concussion symptom severity and recovery.^90^

### Section 3—limitations

One significant limitation of this review was the lack of control groups in 6 of the included studies. Because there was considerable variation in study duration and postinjury interval, it was difficult to compare across mTBI groups. Therefore, the inclusion of a control group was essential for evaluating the relationship between sleep and mTBI across studies. It is also important to note that inclusion and exclusion criteria were not standardized across studies. For instance, some studies relied on self-reports of concussion-like symptoms rather than a physician diagnosis, potentially leading to misclassification of TBI severity. Further, in most studies, participants were not screened for baseline neuropsychiatric or sleep disorders or medications, all of which can impact sleep metrics. No study specifically screened participants for sleep apnea or sleep-disordered breathing, which can have a significant impact on sleep quality. Only Raikes (2016),^70^ Sufrinko (2018),^71^ Hoffman (2020),^75^ and Stevens (2023)^84^ excluded participants for sleep disorders, but these sleep disorders were self-reported rather than screened for. Given the proportion of undiagnosed sleep disordered breathing in this population,^91^ future studies should include specific screening criteria for ruling out—and potentially treating—the presence of sleep apnea.

Another limitation we encountered was a lack of reported adherence data, particularly concerning actigraphy studies. Inconsistent adherence and wear guidelines between studies may have contributed to the variability in results, particularly among those studies with study durations less than 1 week. This is especially relevant among school age populations where weekday and weekend activity and sleep patterns are very different.^92^ Interestingly, not every study reported the use of a sleep diary or sleep log, which could have led to overestimation of sleep time, particularly in the less activity mTBI groups. Finally, while we treated actigraphy and PSG studies similarly in our analyses because there were so few PSG studies, there are distinct differences in these tools that could limit comparisons between the studies. While actigraphy relies on wrist immobility to denote sleep onset, PSG relies on physiological changes in brain electrical activity, which often occurs later, to mark sleep. This could lead leading to a possible overestimation of TST among actigraphy studies and may explain some of the variability in those data.^93^ While Mantua^3^ did report TST on the lower end of the range, Stevens’s^84^ TST estimates were well-within the expected range. Therefore, we do not believe that comparing PSG and actigraphy studies was a major limitation of our analyses.

Another limitation of these studies is the lack of baseline objective sleep values. Given the unpredictable nature of these injuries, it is well-accepted in the mTBI field to use a nonconcussed control group rather than designing a self-controlled study with baseline and postinjury measurements. However, baseline differences in activity levels or sleep metrics could explain a significant portion of the variability between studies. Because this age group can contain significant variation in sleep patterns between individuals, it is particularly important to add some control for baseline sleep characteristics. Future studies should try to control these baseline differences using subjective or objective measures of sleep timing and sleep quality.

## Discussion

### Summary of findings and implications

The purpose of this systematic review was to evaluate changes in sleep following mTBI and identify current gaps in research. We chose to focus on a younger population because (1) they experience more frequent mTBIs, (2) they have fewer age-related comorbidities that can also impact sleep, and (3) adolescence is a critical period for brain development, and sleep disruptions during this time can have long-term impacts on brain health. Studying the relationship between mTBI and sleep is also valuable, as it presents a modifiable avenue for intervention. Importantly, some studies report that improving sleep through cognitive behavioral therapy and blue light therapy can reduce postconcussion symptoms such as fatigue, sleep fragmentation, and depression,^94,95^ suggesting that sleep is a modifiable risk factor in the recovery process.

In general, the results indicated consistent increases in WASO, or sleep fragmentation, across all phases of mTBI recovery, suggesting this may be a reliable metric for detecting mTBI. Sleep fragmentation refers to repetitive, brief awakenings across a sleep period that contribute to a decrease in TST. When sleep fragmentation persists chronically, it can produce physiological effects similar to chronic sleep deprivation, including impaired cognitive function, mood disturbance, fatigue, daytime sleepiness, as well as a variety of other poor health outcomes.^96,97^ Increased WASO is typically correlated with subjective sleep complaints,^98^ potentially explaining frequently reported subjective sleep problems in this population. Fragmented sleep is also associated with impaired executive function and cognitive performance in adolescents, suggesting WASO may mediate the relationship between mTBI and reported neurocognitive outcomes.^99^ Surprisingly, SE, which typically has an inverse relationship with WASO, did not change consistently across studies. While 2 studies reported lower SE among mTBI participants compared with controls, Stevens^84^ reported increased SE. This variability could be due to technical limitations of wrist-based accelerometry in accurately measuring SE or unaccounted baseline differences contributing to large variability among samples. Notably, neither PSG study, which should be more accurate in measuring SE compared to actigraphy, detected a difference in SE during the acute phase of mTBI recovery. However, Stevens (2023)^84^ did report results from a repeat sleep study several weeks after the initial study that showed improvements in WASO, SE, and SOL. This suggests some objective sleep metrics change across recovery phases, and they could serve as markers of mTBI recovery. Future studies should focus on further clarifying these relationships to identify potential objective sleep markers of injury and recovery.

Interestingly, the PSG studies suggested a potential shift in REM sleep patterns across the mTBI recovery spectrum.^3,84^ In acute recovery, there is decreased time spent in REM sleep, but an increased REM latency, meaning it took longer for individuals to begin their first REM period after initial sleep onset. In more chronic recovery phases, time in REM sleep increased, and the longer REM latency persisted. This shift toward more time spent in REM during chronic recovery phases may be evidence of REM rebound, a compensatory increase in REM sleep that typically follows sleep deprivation and other sleep disturbances.^89^ Interestingly, prolonged REM latency is associated with several biomarkers for Alzheimer’s disease, suggesting a potential link between mTBI, sleep, and neurodegeneration.^100^

### Current gaps—sleep microarchitecture and sleep variability

While not reported by either PSG study, sleep oscillations and their topographic expression may offer an additional avenue for identifying markers of mTBI. Other neurological conditions, including obstructive sleep apnea and Alzheimer’s disease, have been identified with signatures within the power spectra that correlate with disease severity.^101,102^ For example, increased beta power correlates with increasing obstructive sleep apnea severity,^103,104^ whereas decreased NREM sigma power is associated with increased tau deposition and worse cognitive performance in Alzheimer’s disease patients.^105^ Given the frequent sleep and cognitive complaints following mTBI, it is possible that mTBI could cause alterations in sleep oscillations.

Many age-related factors can influence sleep oscillations; therefore, analyzing sleep microarchitecture in younger populations is ideal for understanding the impacts of mTBI without other confounds. Two prior studies of adults (ages 18–45 y/o and 18–60 y/o) with concussions due to various etiologies suggested that mTBI altered spectral power in a manner consistent with sleep disruption.^106^ Rao (2011) focused on power spectral analysis during the acute phase of recovery (<2 weeks postinjury) and found decreased delta power in the first NREM period and increased alpha and beta power in subsequent NREM periods.^101,107^ Khoury (2013)^107^ studied the impacts of mTBI on spectral power roughly 3–10 weeks postinjury, and found decreased delta power and increased beta and gamma power. Together, these studies suggest sleep oscillations can be a powerful diagnostic marker of mTBI, and they might help explain long-term sleep complaints.^106,107^ However, no PSG studies to date have looked exclusively at adolescent populations in the context of mTBI.

Several recent studies have also highlighted the importance of assessing day-to-day variability in sleep metrics, particularly in adolescent populations.^108–110^ While many of these studies used the common analysis technique of averaging 5–7 days of actigraphy data together to produce a mean estimate of sleep metrics, this method overlooks sleep variability. Moreover, sleep metrics may shift drastically in the acute injury phase, and this analysis technique could miss subtle changes in sleep metrics. This also highlights the importance of collecting several days of actigraphy data to accurately capture day-to-day changes in sleep measures. Future studies should employ these types of analyses to track changes in sleep metrics across acute recovery phases to identify potential markers of injury severity and recovery.

### Mapping mTBI pathophysiology through sleep

**FIG. 2.**
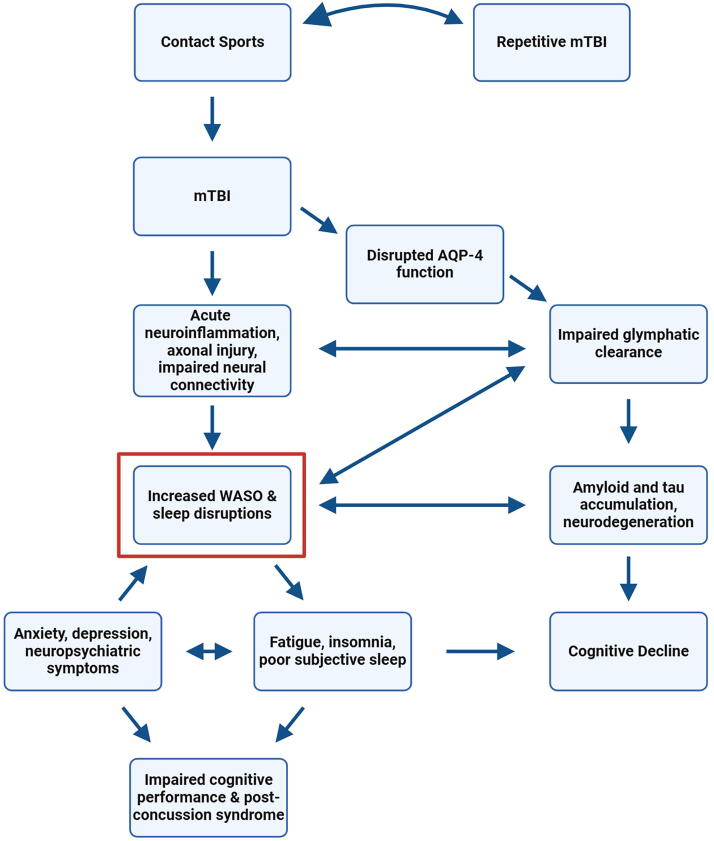
Representative mapping the multidimensional impacts of mTBI. Increased WASO or sleep fragmentation is 1 objective metric identified by actigraphy and PSG studies that could mediate persistent sleep deficits, neuropsychiatric symptoms, and cognitive impairments in adolescent and young adult populations who experience frequent mTBIs. mTBI, mild traumatic brain injury; WASO, wake after sleep onset; PSG, polysomnography.

Our review suggests sleep fragmentation (WASO) is increased across several stages of mTBI recovery. Because WASO can be detected using actigraphy, this metric represents a reliable and inexpensive target for identifying mTBI and potentially tracking recovery. Figure 2 highlights one potential pathway through which sleep disruption may mediate mTBI-associated neuropsychiatric and cognitive symptoms and promote long-term consequences for brain health, such as Alzheimer's disease (AD) and chronic traumatic encephalopathy (CTE). Sleep and AD pathology have a well-established bidirectional relationship, where disturbed sleep increases AD-related pathology, and the presence of AD pathology can further disrupt sleep.^44,105,111–114^ mTBI initiates a neurometabolic cascade involving increased inflammation, as well as subtle damage to tissue, vascular integrity, and network connectivity, which can all cause disrupted sleep.^45,115–117^ Therefore, mTBI-associated chronic sleep disruption may increase risk for AD. One specific consequence of mTBI is dysfunction of the cerebral lymphatic, or glymphatic system.^118–120^ The glymphatic system involves the interchange of cerebrospinal fluid (CSF) and interstitial fluid in the brain parenchyma. CSF influx, facilitated by aquaporin-4 (AQP-4) channels, allows for the convective flux of interstitial fluid, containing solutes and waste such as amyloid and tau, out of the parenchyma, where it is then drained into the cervical lymphatic system, a process vital for brain health.^120–123^ However, mTBI can directly damage AQP-4 function, limiting perivascular efflux and inhibiting waste clearance.^115,117,124^ This impaired glymphatic clearance can synergize with disrupted sleep to increase risk of neurodegenerative disorders, such as AD and CTE, via increased amyloid and tau accumulation.^115,120,125^ In fact, repetitive concussions are linked with CTE and neurocognitive deficits, where earlier exposure to injury predicts worse long-term outcomes.^40,126^ Therefore, this synergistic connection between mTBI and sleep may partially explain long-term brain health consequences.

Individuals with mTBI self-report an increased need for sleep, poor sleep quality, fatigue, and excessive daytime sleepiness well into the first year of recovery.^5^ Independent of mTBI, disrupted AQP-4 function is associated with sleep and mood-related problems, suggesting a mechanistic role of AQP-4 dysfunction in mTBI outcomes (Fig. 2).^51,127,128^ Poor sleep quality increases risk of depression and anxiety, which can feedback to further impair objective sleep metrics.^29,129,130^ Insomnia, another potential sequela of mTBI, is associated with increased risk of dementia and impaired cognitive performance.^29,131^ Lastly, both disrupted sleep and mTBI are associated with inflammatory processes, which can further impair glymphatic clearance and drive AD-related pathogenesis.^132–135^ More work needs to be done to investigate the extent to which mTBI-related sleep disruptions can impair neurocognitive function and lead to long-term brain health risks, however, a clear connection between these factors exists. To best identify markers of mTBI severity and recovery, future studies should aim to connect WASO and other objective measures of sleep to mechanisms involved in this cascade.

### Concluding statement

This review highlighted changes in objective sleep metrics following mTBI in youth and adolescent populations. While results were mixed for several of the metrics, we found increased WASO, suggesting greater sleep fragmentation, across acute and chronic phases of mTBI recovery. Further, we explored how changes in these sleep markers might explain frequently reported sleep and neurocognitive deficits. Future research should focus on long-term studies of young athletes to capture subjective and objective sleep metrics pre- and postinjury. Studying sleep longitudinally can also provide insight into mTBI recovery. Both actigraphy and PSG can be useful tools in identifying changes in sleep, but PSG studies focused on local and global changes in sleep oscillations may provide a greater opportunity for understanding the mechanisms underlying the association between mTBI and sleep. Ultimately, monitoring sleep is a valuable method for evaluating mTBI symptomology. We hope subsequent research will identify specific sleep metrics to target in order to improve recovery timelines and neuropsychiatric outcomes, and design safer return-to-play protocols for young athletes.

## Transparency, Rigor, and Reproducibility Summary

The design for this review followed the most recent PRISMA guidelines for systematic reviews.^65^ Using EndNote software, 3 databases were searched for relevant records, namely, PubMed, Web of Science, and Scopus, and duplicates were removed automatically. Relevant records needed to include “sleep” and “mTBI/mild traumatic brain injury/concussion.” Regardless of relevance, any article published before 2010 was excluded, so this review is not all-encompassing, but rather highlights recent findings in this area of research. The remaining records were manually screened to remove duplicates. Records were then screened for certain key words, to eliminate reviews, animal studies, or studies containing the wrong population, such as “veterans” or “older adults.” The remaining records (*n* = 231) were evaluated by SS and CC to confirm that they (1) contained the correct population (ages 10–30) and (2) included objective metrics of sleep (actigraphy, PSG). Although the keywords “actigraphy” and “polysomnography” could have been used in earlier screening phases, we felt using a comprehensive full-text evaluation would prevent the exclusion of any studies who did not mention these objective metrics in the abstract or title. The final set of 17 articles encompasses all available literature published after 2010 that met our inclusion criteria. Several passes were made by S.S. and C.M.C. before final article selection. The desired age range of participants was 10–30, as we sought to evaluate the effects of mTBI on brain development. We used the mean age of participants to qualify inclusion, meaning that both Barlow (2020),^74^ and Stevens (2023),^84^ were included despite including participants outside of those age ranges. No statistical analysis was performed using parameters in selected articles, and relevant data from each were presented in tabular form and analyzed comparatively across studies. If parameters were averaged by week or days, the weekly or multiday average most proximal to the date of injury was selected. Any differences shown were statistically significant in the corresponding article. We defined the acute period as <2 weeks postinjury, the subacute phase as 2 weeks to 2-months postinjury, and the chronic phase as any study taking place >2 months postinjury. Adolescents were considered between 10–17 y/o, and young adults between 18–25 y/o. Lastly, the final schematic (Fig. 2) is an attempt to contextualize increased WASO findings within what we already know about mTBI-related brain dysfunction. Our model represents several potential mechanistic pathways but given the gaps in our understanding of the long-term consequences of mTBI on adolescent populations, it is likely that several other pathways contribute to these observed deficits. A full categorization of records screened (EndNote 21), broken down into each phase of screening and reasons for exclusion, is available upon request.

## Abbreviations Used

AD: Alzheimer's disease
AQP-4: aquaporin-4
CSF: cerebrospinal fluid
CTE: chronic traumatic enecephalopathy
EEG: electroencephalography
mTBI: mild traumatic brain injury
NREM: non-rapid eye movement
PRISMA: Preferred Reporting Items for Systematic reviews and Meta-Analyses
PSG: polysomnography
REM: rapid eye movement
rmTBI: repeated mild traumatic brain injuries
SE: sleep efficiency
SOL: sleep onset latency
TST: total sleep time
WASO: wake after sleel onset
